# Effect of Different Vaccines and Aluminum Nanoparticles (ALNPs) on Liver and Kidney of Rabbits: Effect Different Vaccine and Alnps on the Liver and Kidney of Rabbit

**DOI:** 10.31661/gmj.v14i.3677

**Published:** 2025-03-20

**Authors:** Mohammed Abdul Hameed Younis, Muhammed Mizher Radhi, Bahaa Fakhri Hussein

**Affiliations:** ^1^ College of Health and Medical Technique, Middle Technical University, Baghdad, Iraq; ^2^ College of Health and Medical Technique, Al Bayan University, Baghdad, Iraq

**Keywords:** Aluminum Nanoparticles, Vaccine, Rabbits, Liver, Kidney

## Abstract

**Background:**

The mechanisms underlying the tissue-specific effects of vaccines and ALNPs remain insufficiently characterized. The present study aimed to investigate recent potential breakthroughs by observing the effect of vaccines and ALNPs on histopathological changes in the kidney and liver.

**Materials and Methods:**

This was an in-vivo study on Thirty male New Zealand white rabbits. The animals were divided into five groups: Control group (group 1, n=6) considered to receive 0.2 ml normal saline injection intramuscularly; while one experimental group (group 2, n=6) was injected ALNPs (Alum-KAl(SO₄)₂·12H₂O) at a low dose (0.05 M) intramuscularly, one group (group 3, n=6) was injected high dose of ALNPs (0.1 M) intramuscularly, one group (group 4, n=6) was injected intramuscular poliomyelitis vaccine (0.2 mL), and last group (group 5, n=6) was injected tetanus, diphtheria, and pertussis vaccine (0.2 mL) intramuscularly. The experiment continued for 30 days (once a day); then kidney and liver were dissected and processed for histopathological analysis.

**Results:**

The kidney showed renal tubular necrosis, and the predominant histopathological finding was glomerular swelling with focal proximal tubule degeneration and mild to severe distorted glomerulus, in addition to exudate edema and intertubular hemorrhage. Meanwhile, in the liver, changes were observed ranging from mild hepatocyte congestion to hepatic sinusoid and portal triad congestion. Anisokaryosis, nuclear vesiculation, binucleation, cytoplasmic inclusions, cytoplasmic swelling, hydropic degeneration, and necrosis were the primary alterations observed in the hepatocytes.

**Conclusion:**

Therefore, the pathological effects of ALNP and vaccines on the liver and kidney were prominent, but more significant changes were observed in ALNP-treated tissue than in vaccine-treated tissues.

## Introduction

Prophylactic vaccines have played an important role in controlling infectious diseases, with successes like smallpox eradication and near-elimination of polio [1]. While vaccines have drastically reduced infectious diseases, but modern subunit vaccines require adjuvants to enhance immunogenicity [2]. Vaccine adjuvants have evolved from traditional alum to advanced nano formulations [3].

Aluminum adjuvants are the most widely used immunotherapies in vaccines. Aluminum salts (Al) are the primary adjuvants in clinical trials and human vaccines, but only in acceptable proportions. Aluminum-based adjuvants like hydroxide (AH) and phosphate (AP) exhibit different absorption rates and tissue distribution patterns in the body [4]. A key property of aluminum compounds is their increased solubility, which generates Al3+ in the periphery. This is positively correlated with increased cell mortality in vitro and may lead to a greater inflammatory response at the injection site [5]. The use of aluminum concentrations must stay within permissible limits to avoid exceeding toxicity thresholds, and approved guidelines regulate their use [6]. Infants and young children are frequently exposed to these compounds in vaccines, which may affect brain development. Advanced research highlights the negative effects of aluminum compounds on biological functions, particularly in neurological and autoimmune disorders [7]. While engineered aluminum-based nanoparticles in therapeutic cancer vaccines demonstrate minimal cytotoxicity in neural cells, soluble aluminum ions have been shown to induce mitochondrial damage and apoptosis [8]. Additionally, studies comparing aluminum hydroxide nanoparticles to bulk aluminum hydroxide in neonatal mice reveal that nanoparticle exposure results in less severe tissue damage and systemic inflammation [9]. As the current literature on aluminum nanoparticles (ALNPs) and vaccines has primarily focused on their neurotoxic effects, with limited attention to their potential impact on renal and hepatic histopathology, we aimed to investigate the histopathological changes in the kidney and liver of rabbits exposed to ALNPs and inactivated vaccines. This study is novel in that it shifts the focus from the well-studied neurotoxicity of ALNPs to their potential renal and hepatic effects, comparing them with conventional vaccines.

## Materials and Methods

**Figure-1:**
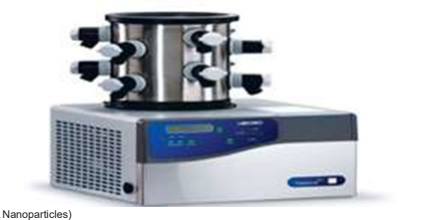
Figure 1. FESEM (AL Nanoparticles)

**Figure-2:**
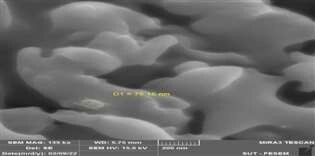
Figure 2. AFM of (AL NPs)

**Figure-3:**
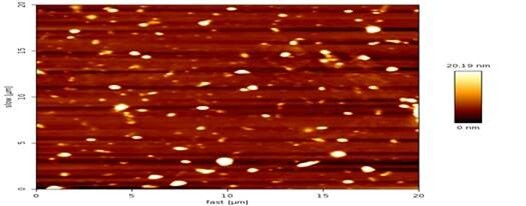
Figure 3. AFM of (AL NPs)

### Materials

This was an in-vivo study that was conducted in compliance with the ARRIVE guidelines at Middle Technical University (MTU) of Baghdad, in April-May 2023. Ethics of work with animals were complied with anesthesia and euthanasia to minimize suffering while maintaining minimal sample sizes (n=30) for statistical validity.

Alum KAl(SO4)2.12H2O was used in the study from a Chinese company (Sinopharm Chemical Reagent Co., Ltd. Catalog No.: 7784-24-9), inactivated poliomyelitis vaccine from Bilthoven Biologicals (Netherlands), BCG, Diphtheria Tetanus pertussis, Type B conjugate vaccine from Shameerpet, Medchal-Malkajgiri District, Telangana state, India. The blood samples were obtained from the rabbit’s heart following administration of various vaccinations and differing doses of AL NPs.

Thirty male New Zealand white rabbits weighing between 2000 and 3200 grams, all of the same age, were used in the study. The animals were housed under standard pathogen-free (SPF) conditions in ventilated cages with sterile bedding. The environment was maintained at a controlled temperature (22 ± 2°C) with a 12-hour light/dark cycle, and the rabbits had ad libitum access to food and water.

### Experimental Groups

The rabbits were randomly (by drawing labeled cards from a shuffled deck) divided into five groups (six per group) as follows:

Group 1 (Control): Injected with 0.2 mL normal saline intramuscularly.

Group 2 (Low-dose ALNPs): Injected with 0.05 M aluminum nanoparticles (ALNPs) intramuscularly.

Group 3 (High-dose ALNPs): Injected with 0.1 M ALNPs intramuscularly.

Group 4: Injected with 0.2 mL inactivated poliomyelitis vaccine intramuscularly.

Group 5: Injected with 0.2 mL tetanus, diphtheria, and pertussis (DTP) vaccine intramuscularly.

The experiment lasted for 30 days, once a day. At the end of the study, the animals were anesthetized using ketamine (30 mg/kg) and xylazine (3 mg/kg) via intramuscular injection before being sacrificed. Anesthesia was confirmed by the absence of pedal and corneal reflexes before proceeding with the sacrifice. Tissue samples (liver and kidneys) were collected from all groups for histological examination. Small fragments of each tissue were fixed in 10% neutral buffered formalin. After several days of preservation, the tissues were processed for microscopic analysis. Standard histological procedures, including hematoxylin and eosin (H&E) staining, were performed as described by Bancroft and Gamble (2002) [10].

Preparation and Characterization of Aluminum Nanoparticles (ALNPs):

Micro-sized alum was converted into nanoparticles using lyophilization, with the lyophilizer (Labconco FreeZone 4.5-Liter Benchtop Freeze Dryers, made in US). The resulting alum nanoparticles were characterized using spectroscopic techniques, including atomic force microscopy (AFM) and field emission scanning electron microscopy (FESEM), as shown in Figures-1and2, respectively.

FESEM analysis confirmed the size (~75.15 nm) and shape of the alum nanoparticles (Figure-2), consistent with findings reported by Pandey and Dahiya (2016) [11]. AFM analysis further demonstrated the spherical morphology of the alum nanoparticles (Figure-3).

### Outcomes

Key outcomes included significant alterations in hepatic architecture, such as Kupffer cell proliferation, nuclear abnormalities (anisonucleosis, pyknosis, and vesiculation), and cytoplasmic changes (eosinophilia, Mallory hyaline-like inclusions). Renal damage was also observed, including glomerular distortion, tubular atrophy, interstitial hemorrhage, and acute tubular necrosis.

## Statistical Analysis

Data were compared among groups qualitatively as low sample size did not allow statistical comparison.

## Results

**Figure-4:**
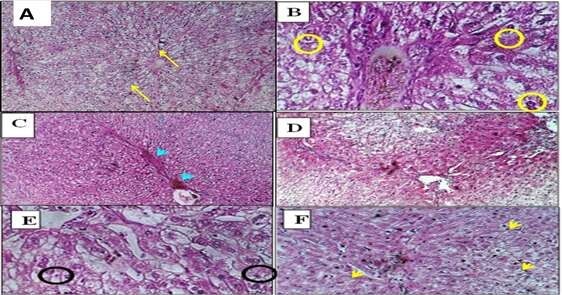
Figure 4. Histopathology of liver sections from male rabbits treated with low and high doses of ALNPs. (A) Control group showing normal liver architecture (H&E, ×100). (B) Hepatocytes displaying anisonucleosis (H &E, ×400). (C) Nuclear vesiculation (H&E, ×100). (D) Nuclear pyknosis (H&E, ×100). (E) Binucleation in low-dose ALNP group with increased Kupffer cell prominence. (F) Autolytic changes (H&E, ×400).

**Figure-5:**
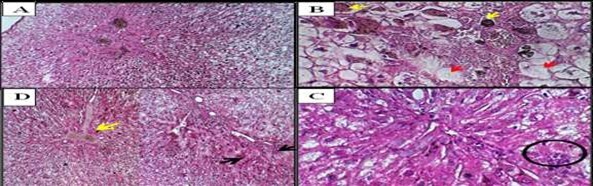
Figure 5. Liver sections from vaccine-treated rabbits. (A) Focal hepatic necrosis (H&E, ×100). (B) Hydropic degeneration (red arrows) and severe necrosis (yellow arrows) (H&E, ×400). (C) Multinucleated giant cells (black circle) adjacent to mononuclear hepatocytes (H&E, ×400). (D) Hepatocellular necrosis (black arrows) and suppurative cholangitis (yellow arrow) (H&E, ×100).

**Figure-6:**
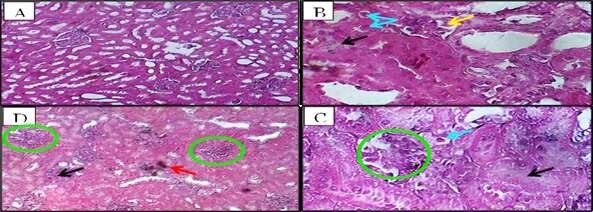
Figure 6. Kidney histopathology in ALNP-treated groups. (A) Normal control. (B) Low-dose ALNP showing glomerular distortion (yellow arrow), edema (black arrows), and tubular degeneration with pyknotic nuclei (blue arrows) (H&E, ×100). (C) High-dose ALNP with distorted glomeruli (green circle) and dilated tubules containing pyknotic nuclei (blue circle) (H&E, ×100). (D) Intertubular hemorrhage (red arrow) (H&E, ×100).

**Figure-7:**
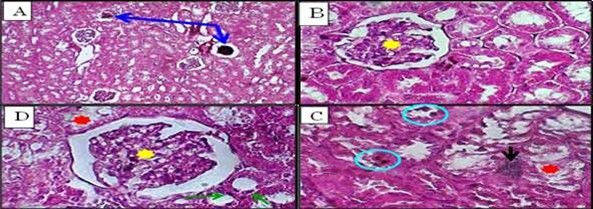
Figure 7. Kidney sections from vaccine-treated rabbits. (A) Vaccine (1) group showing atrophic glomeruli (blue arrows) (H&E, ×100). (B) Distorted glomerulus (yellow star) in vaccine (1) group (H&E, ×400). (C,D) Vaccine (2) group demonstrating intertubular hemorrhage (black arrow), tubular degeneration (green arrows), denuded epithelium (red stars), and focal necrosis (blue circle) (H&E, ×400).

**Table:** Table1. Comparative Histopathological Effects of ALNPs and Vaccines on Liver and Kidney Tissues in Rabbits

**Group**	**Liver Findings**	**Kidney Findings**
**Control (Group 1)**	• Normal lobular architecture (Figure-4A) • Intact hepatocyte cords, Kupffer cells	• No pathological changes
**Low-dose ALNP (Group 2)**	• Kupffer cell proliferation • Mild anisonucleosis (Figure-4B) • Nuclear vesiculation (Figure-4C) • Focal autolysis (Figure-4F)	• Glomerular distortion • Tubular atrophy • Exudative edema (Figure-6B)
**High-dose ALNP (Group 3)**	• Severe Kupffer activation • Pyknosis (Figure-4D), binucleation (Figure-4E) • Mallory hyaline-like inclusions	• Glomerular thickening • Interstitial hemorrhage • Tubular degeneration (Figure-6C, D)
**Poliomyelitis Vaccine (Group 4)**	• Focal necrosis (Figure-5A) • Hydropic degeneration (Figure-5B) • Periportal inflammation	• Acute tubular necrosis • Brush border loss • Tubular dilation (Figure-7A, B)
**DTP Vaccine (Group 5)**	• Syncytial hepatocytes (Figure-5C) • Portal fibrosis, cholangitis (Figure-5D) • Severe necrosis	• Granular casts • Epithelial denudation • Focal apoptosis (Figure-7C, D)

Table-1 describes the main findings of study in each group.

The parenchyma in the lobular liver of control rabbits had a typical architecture characterized by cords extending from the central vein to the portal area (portal triad). Compared to the control group (Figure-4A), hepatocytes were organized in cords interspersed with capillary sinusoids bordered by endothelial cells. Kupffer cells were also located along the sinusoidal capillary. The following histopathological changes were detected in the liver injected with ALNP and vaccine in male rabbits.

Groups with low and high ALNP injections showed greater impact on liver parenchyma. The sinusoidal Kupffer cells became prominent and proliferated; this change was evident early at low doses of ALNP and was more dramatic with greater doses of ALNP injection. Additionally, a small number of hepatocytes showed signs of mild anisonucleosis (Figure-4B). As ALNP exposure increased, this change became obvious, especially at elevated concentrations (Figure-4B). Nuclear vesiculation was first noted in the liver after administering a low dose of ALNP in rabbits, but it became rare or completely stopped with extended ALNP treatment (Figure-4C). Some hepatocytes, particularly those that had died (Figure-4D), showed signs of nuclear pyknosis. Binucleation was observed in rabbits receiving a high dosage of ALNP (Figure-4E). In the cytoplasm of certain hepatocytes, filamentous inclusions that looked like Mallory hyaline bodies were seen; these were more noticeable at higher doses and after longer exposure to ALNP. Particularly in hepatocytes with poorly defined cell membranes, eosinophilic cytoplasm and intermittent autolytic alterations were noted (Figure-4F). This alteration was observed in rabbits administered a low dose of ALNP over an extended duration.

In groups inoculated with vaccines (1) and (2), there was a greater impact on the liver parenchyma compared to those injected with ALNP. Clearly delineated necrosis in the hepatic cells manifested as sporadic focal lesions observed in certain hepatocytes of vaccination (1)-treated rabbits (Figure-5A). A moderate level of hydropic degeneration and cytoplasmic enlargement of the hepatocytes was observed, with an increase in severity associated with necrosis in the male rabbits following the second vaccine administration (Figure-5B). The vaccinated group also showed signs of a necro-inflammatory reaction around the portal (2). The isolated cell necrosis primarily presented as intercellular spherical eosinophilic structures (apoptotic bodies) surrounded by clear vacuoles. The hepatocytes in the periportal regions demonstrated increased vulnerability to necrosis. Hepatic vaccination also showed multinucleated syncytial hepatocytes [2], which were mostly located in the liver and appeared as single clusters of big cells or multinucleated huge cells sitting on top of mononuclear hepatocytes (Figure-5C). Hepatocellular necrosis, portal inflammation (mainly lymphocytic but also including plasma cells and neutrophils), periportal fibrosis, cirrhosis, and bile duct lesions (including suppurative destructive cholangitis, periductal scarring, and ductopenia) were among the histological findings (Figure-5D).

In the kidney, interstitial and intraglomerular congestion or tubular atrophy changes were observed in the groups injected with low and high doses of ALNP. In both the low and high ALNP groups, the kidney cortex revealed glomerular deterioration along with Bowman’s capsule dilatation and cylindrical cell degeneration with pyknotic cores (Figure-6D, B, and C). Additionally, localized rounded injury associated with misshaped glomeruli and a thickened capsule in the group injected with high ALNP (Figure-6C, D), along with an area of exudate edema and intertubular hemorrhage (Figure-6).

The first two vaccines showed abnormalities such as acute tubular necrosis, widespread or patchy denudation of the renal tubular cells, and loss of the brush border, in addition to the flattening of the renal tubular cells due to tubular dilation (Figure-7A, B). In vaccine (2)-injected groups, intratubular casts were observed and different types of sloughing cells, which are responsible for the formation of granular casts. Even though the cellular debris and denuded epithelium made the intratubular blockage obvious, the clumping of the denuded tubular epithelial cells was due to a reorganization of intercellular adhesion molecules (Figure-7C, D). Subsequently, there was diminished and distorted glomeruli and dilated tubules. Also observed was a variable extent of renal tubular necrosis/apoptosis with focal degeneration (Figure-7C).

## Discussion

We studied the effect of ALNP and vaccine in male rabbits. Microscopically, in the liver injected with ALNP, we observed slightly to moderately dilated sinusoids with sinusoidal Kupffer cells, which increased as shown in Figure-4B. With high-dose ALNP, we found mild anisonucleosis in hepatocytes (Figure-4B). Also, nuclear vesiculation was evident at both low and high ALNP doses, as shown in Figure-4C. Nuclei appeared pyknotic (Figure-4D) and binucleated in the high-dose ALNP group (Figure-4E). Another finding was the presence of filamentous inclusions in hepatocyte cytoplasm, which resembled Mallory hyaline bodies. These changes increased with dose and time (Figure-4F). Figure-4 shows well-defined necrosis of hepatic cells, hydropic degeneration, cytoplasmic swelling, and apoptosis in male rabbits administered vaccines.

Kupffer cells increase in number to detoxify blood (Bashir and Noory, 2012 [12]). The presence of inflammatory cell infiltrate in the liver suggests that ALNP could lead to reactive oxygen species (ROS) generation, as described by Johar et al. (2004) [13]. Concerning nuclei, Zusman et al. (1991) [14] found nuclear polymorphism in hepatic dysplasia and carcinoma. Binucleated cells indicate chromosome hyperplasia during cell regeneration [15][16]. Other changes in hepatic cells induced by vaccines may result from mitochondrial swelling, endoplasmic reticulum edema, and lysosomal rupture, as described by Jarrar and Taib (2012) [16]. Kupffer cells, which are macrophages in the liver, also store nanoparticles up to 100 nm in size [17]. Through renal filtration, particles ≤5 nm are quickly removed from circulation [18]. On the other hand, metal nanoparticles (MeNPs) have been found to influence innate immunity in some studies. In vitro studies showed MeNPs have cytotoxic and genotoxic effects, altering receptors involved in gene expression and cytokine production. Inflammation and the release of cytokines, chemokines, and free radicals such as nitric oxide mediate the innate immune response to threats [19].

In rabbits injected with ALNP (low and high) or vaccines (1 and 2), glomerular degeneration, Bowman’s capsule dilatation, and tubular cell degeneration with pyknotic nuclei were observed. The small size of vaccine components allows them to pass through the glomerular barrier and be taken up by renal tubular cells. These substances cause damage primarily in the cortical region before reaching the proximal tubule, progressively impairing kidney function. Another mechanism may involve free radical accumulation in renal tissues due to ALNP-induced lipid peroxidation. In cadmium-exposed rats, cadmium accumulates in the kidney, impairing glomerular filtration [20][21]. Vaccines and fluid imbalance can cause intracellular water accumulation, promoting vacuolar degeneration in kidney tissue [1][2]. While kidney degeneration and necrosis may arise from immune resistance to foreign particles, intoxication, hemodynamic changes, or altered inflammatory/apoptotic genes, other lesions reflect kidney clearance of these substances. Further research is needed to determine the exact mechanisms [22][23].

### Recommendation

Based on the observed hepatorenal toxicity, future studies should: (1) investigate smaller ALNP doses and surface modifications to reduce tissue damage, (2) evaluate long-term effects of vaccine-adjuvant combinations, and (3) explore chelating agents or antioxidants to mitigate nanoparticle-induced oxidative stress.

## Conclusion

This study demonstrated the effect of Vaccine [inactivated poliomyelitis vaccine (1), BCG, Diphtheria Tetanus pertussis (2)] and ALNPs [Alum KAl (SO4)2.12H2O] on the liver and kidney. Moreover, the present study aimed including recent potential breakthroughs, while the interaction of Vaccine and ALNPs with proteins of cell tissue was observed from the histopathological changes, showing that Vaccine [1][2] and ALNPs may be considered as materials possess a side effecting on the liver and kidney. However, further chronic studies are needed to explore mechanisms by which the Vaccine and ALNPs to these bad effects at this tissue.

## Conflict of Interest

None.
